# *Streptomyces lunalinharesii* Strain 235 Shows the Potential to Inhibit Bacteria Involved in Biocorrosion Processes

**DOI:** 10.1155/2013/309769

**Published:** 2013-01-28

**Authors:** Juliana Pacheco da Rosa, Elisa Korenblum, Marcella Novaes Franco-Cirigliano, Fernanda Abreu, Ulysses Lins, Rosângela M. A. Soares, Andrew Macrae, Lucy Seldin, Rosalie R. R. Coelho

**Affiliations:** Departamento de Microbiologia Geral, Instituto de Microbiologia Prof. Paulo de Góes, Centro de Ciências da Saúde, Universidade Federal do Rio de Janeiro, Bloco I, Ilha do Fundão, 21941-590 Rio de Janeiro, RJ, Brazil

## Abstract

Four actinomycete strains previously isolated from Brazilian soils were tested for their antimicrobial activity against *Bacillus pumilus* LF-4 and *Desulfovibrio alaskensis* NCIMB 13491, bacteria that are well known to be involved in biofilm formation and biocorrosion. Strain 235, belonging to the species *Streptomyces lunalinharesii*, inhibited the growth of both bacteria. The antimicrobial activity was seen over a wide range of pH, and after treatment with several chemicals and heat but not with proteinase K and trypsin. The antimicrobial substances present in the concentrated supernatant from growth media were partially characterized by SDS-PAGE and extracellular polypeptides were seen. Bands in the size range of 12 to 14.4 kDa caused antimicrobial activity. Transmission electron microscopy of *D. alaskensis* cells treated with the concentrated supernatant containing the antimicrobial substances revealed the formation of prominent bubbles, the spherical double-layered structures on the cell membrane, and the periplasmic space completely filled with electron-dense material. This is the first report on the production of antimicrobial substances by actinomycetes against bacteria involved in biocorrosion processes, and these findings may be of great relevance as an alternative source of biocides to those currently employed in the petroleum industry.

## 1. Introduction

Corrosion is a leading cause of pipe failure and high maintenance costs in gas pipelines [1]. Biocorrosion is defined as a corrosive damage initiated or aggravated by the direct or indirect activities of microorganisms [2]. A wide range of bacteria are present in most if not all areas of oil production and have been described from water injection plants, drilling mud, and live reservoir cores [3–6]. Among the aerobic and anaerobic bacteria related to biocorrosion in oil industries, the sulfate-reducing bacteria (SRB) have been extensively studied because as well as forming corrosive biofilms they also produce hydrogen sulfide [3], which can result in health risks to workers [7]. A variety of *Bacillus* species have been shown to form biofilms on metal surfaces and produce elaborated multicellular communities that display conspicuous architectural features [8]. Jack et al. [9] demonstrated a significant increase in corrosion rates of carbon steel in a continuously flowing freshwater reactor where a biofilm of *Bacillus* and SRB had been observed. 

The most common method for controlling microbial growth in industrial water systems is the use of biocides [10]. Oxidizing (chlorine, ozone) or nonoxidizing compounds (quaternary ammonium salts, aldehydes, and tetrakis (hydroxymethyl)phosphonium sulfate—THPS) are commonly applied [11, 12]. However, the environmental impact and cost of adding large quantities of these compounds must also be considered when such biocides are used [13]. So, the use of biocides of a microbial nature and origin offers another option. Indeed, the production of antimicrobial substances (AMSs) able to inhibit SRB growth has already been shown as an attractive alternative to the use of biocides [5, 13]. 

Actinomycetes are well known as potent producers of a variety of secondary metabolites with distinct biological activities [14, 15], including AMSs active against both pathogenic [16–18] and phytopathogenic microorganisms [18–20]. However, the production of AMSs active against bacteria involved in biocorrosion process has been poorly described thus far. The exploration of soils and other habitats for microbes of biotechnological interest has led to the isolation of novel actinomycete strains [21]. Brazilian soils offer great potential for bioprospection for novel strains and new bioactive compounds. In previous studies, we have isolated several actinomycete strains from these soils. Therefore, the search among these strains for those with antimicrobial properties against microorganisms that negatively impact on the oil industry was likely to reveal a strain with biotechnology potential.

In this study four different strains of actinomycetes, previously isolated from Brazilian tropical soils and previously selected as promising for AMS production, had their ability to produce AMSs against the *Bacillus pumilus* LF-4 and the *Desulfovibrio alaskensis* NCIMB 13491 evaluated. In this paper, one strain, identified as belonging to the *Streptomyces lunalinharesii* species, is reported and discussed. A preliminary characterization of the AMS obtained from the culture supernatant and its effect on SRB cells are also described. The antimicrobial substance produced by *S. lunalinharesii *235 might be an important alternative biocide for use against undesirable bacteria that colonize pipe and flow lines used in the production of oil and gas.

## 2. Material and Methods

### 2.1. Bacterial Strains and Growth Conditions

The actinomycete strains used in this study were originally isolated from Brazilian tropical soils prior to 2001. The genus and the origin of these bacterial strains are presented in Table 1. They were previously selected as promising for the production of AMSs against different microbial strains [22–25] and now were used to test their capability to produce AMSs with antimicrobial activity against aerobic and anaerobic bacteria associated with biocorrosion processes. Unless otherwise stated, these strains were grown in yeast extract-malt extract-agar (YMA) [26] under aerobic conditions at 28°C for 7 days. 

The *Bacillus pumilus* LF-4 and the SRB *Desulfovibrio alaskensis* NCIMB 13491 were used as indicator strains for the production of AMSs*. B. pumilus *LF-4 was originally isolated from an oil reservoir in Brazil located in a deep-water production basin at an off-shore platform in Rio de Janeiro [27]. It was grown under aerobic conditions for 24 h at 30°C, in the Luria-Bertani broth (LB) [28]. The SRB strain was isolated from a soured oil reservoir [29] and grown in Postgate C medium [30] at 30°C for 3 days, in anaerobic conditions using sealed serum bottles (10 mL). The bottles were purged with a N_2_ flux to achieve anaerobiosis. All the bacterial strains were maintained in a long-term storage by freezing the cells at −20°C in 20% glycerol.

### 2.2. Antimicrobial Activity Assay

For testing the actinomycete strains, the overlay method was performed, as described by Rosado and Seldin [31]. All strains were spot inoculated (5 *μ*L) on the surface of YMA plates, and after incubation at 28°C for 7 days, they were killed by exposure to chloroform vapors for 15 min. The plates were then flooded with either *B. pumilus* LF-4 or *D. alaskensis* and incubated at 30°C for 24 h or 5 days, respectively. Manipulation of *D. alaskensis* was performed in an anaerobic chamber (Plas Labs, Lansing, MI, USA). Clear inhibition zones around the spot inoculum indicated antimicrobial production. The diameters of the inhibition zones were scored as follows: (−) no inhibition, (+) weak inhibition with clear zones <7 mm, (++) moderate inhibition with clear zones between 7 and 12 mm, and (+++) strong inhibition with clear zones >12 mm [32].

To test the antimicrobial activity of the supernatants, 20 *μ*L aliquots were spotted on solid LB media containing a growth lawn of *B. pumilus* LF-4. Plates were maintained at 30°C/24 h and then inhibition zones were observed and their size recorded. 

### 2.3. Influence of Growth Conditions on the AMS Production

The overlay method was also performed using the actinomycete strains grown in two chemically defined agar media [32], containing a mineral salt solution and either glucose or glycerol as a carbon source. After incubation in aerobic conditions at 28°C for 7 days, the antimicrobial activity of strains was tested against *B. pumilus *LF-4.

The influence of pH and aeration on AMS production was tested in liquid medium using the chemically defined medium containing glucose [32]. Four different pH values (5.0, 6.0, 7.0, and 8.0) and two aeration conditions (stationary or shaking, 200 rpm) were tested. After incubation at 28°C for 7 days, each culture was filtered with filter Whatman paper no. 1, and the supernatants were lyophilized for further analysis. The lyophilized supernatants were concentrated 100-fold and 300-fold. The antimicrobial activity of each concentrated supernatant was tested as described above.

### 2.4. Molecular Identification of *Streptomyces* sp. 235

After growth of *Streptomyces* sp. 235 in YMA for four days at 28°C with agitation (200 rpm), genomic DNA was extracted as described in earlier reports [33]. PCR amplification of the *rrs* gene was performed using a GoTaq Green Master Mix Kit (Promega Corporation) according to the manufacturer's instructions. The amplification was carried out using the pair of universal primers 27F [34] and 1541R [35]. The PCR amplification conditions were 35 cycles of 95°C (30 s), 55°C (30 s), and 72°C (50 s) in a thermal cycler model Gene Amp PCR System 9700 (Applied Biosystems). A hot start (5 min at 95°C) was applied to avoid initial mispriming and enhance primer specificity. A final extension step was run for 7 min at 72°C and the reaction tubes were then cooled to 4°C. Amplified fragments were purified using the Illustra GFX PCR DNA and Gel Band Purification Kit (GE Healthcare), which was used according to the manufacturer's instructions. A negative control (without DNA) was run in all amplifications. DNA preparation and PCR products were visualized after electrophoresis in 1x TBE buffer on a 1.2% agarose gel [36]. The purification product was sequenced by the Center for Human Genome Studies at the University of São Paulo, Brazil. All sequences were identified using BLAST [37] at the National Center for Biotechnology Information (http://www.ncbi.nlm.nih.gov/blast). Sequences retrieved were aligned with the most similar-type strains obtained using CLUSTAL X [38]. BioEdit v. 7.0.0 (http://www.mbio.ncsu.edu/Bioedit/bioedit.html) was used for manual editing of the sequences and a phylogenetic tree with 1000 bootstrap replicates was constructed using MEGA 4 software (http://www.megasoftware.net/). The sequence obtained was deposited in the GenBank database under accession number GU126551. 

Genomic DNA-DNA hybridization experiments were performed to compare strain 235 with its closest-type strain relative as determined by 16S rRNA gene sequences. Levels of genomic DNA-DNA similarity were determined at the Deutsche Sammlung von Mikroorganismen und Zellkulturen GmbH (DSMZ). Cells were disrupted by using a French pressure cell (Thermo Spectronic) and the DNA in the crude lysate was purified by chromatography on hydroxyapatite [39]. DNA-DNA hybridization was carried out as described by De Ley et al. [40] under consideration of the modifications described by Huss et al. [41] using a model Cary 100 Bio UV/VIS spectrophotometer equipped with a Peltier-thermostatted 6X6 multicell changer and a temperature controller with *in situ* temperature probe (Varian).

### 2.5. Partial Characterization of AMSs Produced by Strain 235

Partial characterization was performed using a concentrated supernatant containing the AMSs, obtained from growth of the strain 235 in the liquid medium containing glucose at pH 7.0 [32]. After incubation at 28°C for 7 days, the culture was filtered with Whatman paper no. 1, and the supernatant lyophilized and 300-fold concentrated in the same chemically defined medium.

To estimate the molecular mass of AMSs the concentrated supernatant (500 *μ*L) was submitted to centrifugation at 4°C and 3 000 rpm for 1 h in an ultrafiltration membrane (Millipore Corp., USA) with a 10 000 Da molecular mass cut off. Both fractions, the concentrated one (MM > 10 000 Da) and the excluded one (MM < 10 000 Da), were tested for antimicrobial activity against *B. pumilus* LF-4 in LB plates. 

The effect of organic solvents, chemicals, proteolytic enzymes, heat and pH on activity of AMSs was tested (Table 2) using 20 *μ*L of the concentrated supernatant. Systems containing the concentrated supernatant and equal volume of each solvent, chemical, or enzyme were incubated for 2 hours at room temperature (for organic solvents and chemicals) or at 37°C (for enzymes). For the heat treatment, the concentrated supernatant was incubated during 45 or 60 min, at 40, 60, 80, and 100°C, or autoclaved (121°C for 20 min). For pH stability, the concentrated supernatants were mixed to the same volume of citric acid-sodium citrate buffer to achieve different pH values lower than 6.0 and with Tris-HCl buffer for pH higher than 7.0. Antimicrobial activities against *B. pumilus* LF-4 were determined before and after all treatments.

### 2.6. Sodium Dodecyl Sulfate-Polyacrylamide Gel Electrophoresis (SDS-PAGE)

Ten microliters of the same 300-fold concentrated supernatant described above were treated with equal volume of SDS-PAGE sample buffer (125 mM Tris-HCl, pH 6.8, 4% SDS, 20% glycerol, 2% mercaptoethanol, 2% bromophenol blue). Proteins were analyzed in 20–5% gradient SDS-PAGE by the method described by Laemmli [42]. Following electrophoresis conducted at 100 V at 4°C for 140 min, each lane of the gel was cut vertically. One lane was stained with 0.025% Comassie brilliant blue R-250 in methanol-acetic acid-water (40 : 7 : 53) and destained in the same solvent, to determine the secretory protein profile. The apparent molecular masses of the polypeptides were calculated by comparison with the mobility of Full-Range Rainbow Molecular Weight Markers (GE Healthcare, Buckinghamshire, UK). To localize the *in situ* antimicrobial activity, a bioautography was performed: the other lane was prewashed with 10 volumes of 1% Triton X-100 in water for 1 h at room temperature, under agitation, to remove the SDS. Then, the gel strips were washed three times (20 min each) with double-distilled water, transferred to a LB agar plate, and overlaid with 4 mL semisolid (0.6%) LB containing 0.4 mL of the indicator strain (*B. pumilus *LF-4). Plates were then incubated in aerobic conditions at 30°C for 24 h and examined for the presence of an inhibitory zone.

### 2.7. Inhibitory Effect of AMSs on *D. alaskensis*

The inhibitory effect of AMSs on* D. alaskensis* was assessed by the determination of the minimal inhibitory concentration (MIC) and/or the minimal bactericide concentration (MBC). The MIC was performed using a microdilution method. The SRB were grown in liquid Postgate C medium for about 24–48 h at 30°C under anaerobic conditions. Experiments were performed using a 96-well plate, where each well contained 100 *μ*L of SRB suspension (10^7^ cells/mL), and suspensions were exposed to different amounts of AMSs (from 0.1 to 0.003 g protein/mL) diluted in Postgate C medium, in order to obtain successive dilutions of AMSs (from 1/1, 1/2, 1/4, 1/8, 1/16, until 1/2032) and a final volume of 200 *μ*L. Experimental controls included a medium without AMSs or a medium plus cell suspensions without AMSs. After incubation at 30°C for 7 days, growth of *D. alaskensis* was confirmed visually, by the observation of a blackish precipitate (FeS, from the reaction of H_2_S, produced by the SRB cells, and Fe, present in the medium), and then measured spectrophotometrically at 630 nm. The MIC was defined as the lowest amount of AMSs required to ensure that SRB growth was absent.

To perform the MBC test, aliquots of 10 *μ*L from the wells where growth was absent in the MIC test were used to inoculate fresh Postgate C medium (90 *μ*L). The same controls were used, and after incubation for 7 days at 30°C, the MBC was determined as the lowest concentration where growth was absent. All the inoculation procedures and incubations in MIC and MBC tests were performed in an anaerobic chamber (Plas Labs, Lansing, MI, USA) and the experiments conducted five times. Protein determination was according to Bradford [43].

### 2.8. Transmission Electron Microscopy (TEM)

TEM was performed to examine the ultrastructural changes in the *D. alaskensis* cells treated with MIC, sub-MIC, and supra-MIC of AMSs during 7 days. Postgate C medium was also tested as a negative control. The mixtures were harvested by centrifugation at 4 000 ×g for 15 min and the pellet washed three times in a reducing solution (0.0124% sodium thioglycollate, 0.01% ascorbic acid, and 4 mL of a solution of 0.025% resazurin). Then cells were fixed for 2 h, at room temperature, with 2.5% glutaraldehyde and 4% paraformaldehyde in 0.1 M cacodylate buffer, pH 7.2. Postfixation was carried out in 1 % osmium tetroxide in 0.1 M cacodylate buffer for 1 h. Cells were further dehydrated in an acetone series and embedded in PolyBed 812. Ultrathin sections were obtained using a Leica ultramicrotome, stained with uranyl acetate and lead citrate, and observed in a FEI Morgagni TEM at 80 kV.

## 3. Results

### 3.1. Antimicrobial Activity Assay

**Figure 1:**
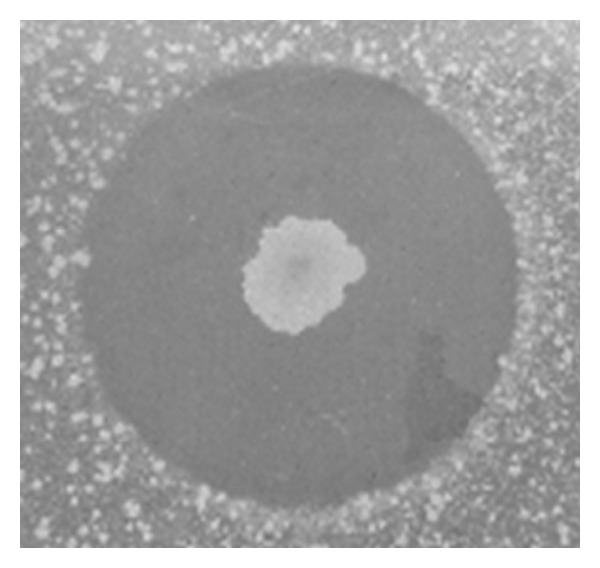
Inhibition zone of *D. alaskensis *NCIMB 13491 by *S. lunalinharesii* 235 on agar plate.

All four actinomycete strains (Table 1) were able to inhibit aerobic *B. pumilus* LF-4 and anaerobic *D. alaskensis* when tested using the overlay method in YMA medium.  Figure 1 shows the inhibition of *D. alaskensis* by strain 235, where a 12 mm inhibition zone was observed. 

### 3.2. Influence of Growth Conditions on the AMS Production and Strain Selection

All four strains were also able to inhibit *B. pumilus* LF-4 grown on solid media with either glucose or glycerol as a carbon source. Strains 221 and 235 were more antagonistic on solid media and were selected for further studies in liquid media. Strain 221 supernatants failed to inhibit *B. pumilus *LF-4 at less than a 400-fold concentration. Given that the 300-fold concentrated supernatant from strain 235 did show inhibitory activity, strain 235 was chosen for identification and further studies.

### 3.3. Molecular Identification of *Streptomyces* sp. 235

**Figure 2:**
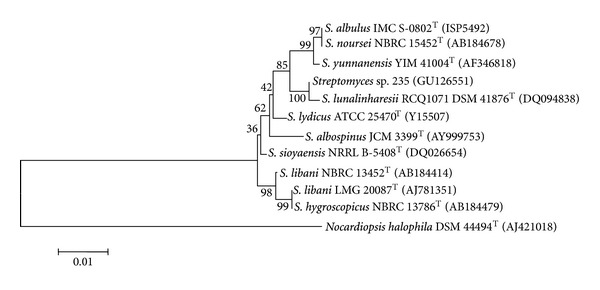
Phylogenetic tree obtained by the neighbor-joining method, based on the alignment of 16S rDNA of strain 235 and other *Streptomyces* species. *Nocardiopsis halophila* was used as an out-group. Bootstrap analyses were performed with 1000 repetitions. The scale bar corresponds to 0.01 substitutions per nucleotide position.

The identification of strain 235 was carried out by PCR amplification of *rrs* gene (1498 bp), its sequencing, and then genomic DNA-DNA whole-genome hybridization homology. According to the phylogenetic tree (Figure 2), the most closely related bacterial type strain was *Streptomyces lunalinharesii* RCQ1071 (accession number DSM 41876T) [44], which shares 99.0% similarity within its 16S rDNA gene sequence. Genomic DNA homology between the *Streptomyces lunalinharesii* type strain and 235, analyzed by DNA-DNA hybridization (DSMZ service), demonstrated that these strains belong to the same species.

### 3.4. Partial Characterization of AMSs Produced by *Streptomyces* sp. 235

The AMS that was retained by the ultrafiltration membrane (10 000 Da cutoff) and its characterization can be seen in Table 2. The antimicrobial activity was resistant to pronase E, different chemicals (urea 6 M, NaOH 0.2 and 1 M, and HCl 0.2 M) and organic solvents (acetone, ethanol, methanol, and chloroform at 10 and 50%) but sensitive to proteinase K and trypsin. It was heat stable after incubation at 100°C for 1 h but did not maintain its activity after being autoclaved at 121°C for 20 min. It was active in the range of pH values between 3.0 and 9.0.

### 3.5. Sodium Dodecyl Sulfate-Polyacrylamide Gel Electrophoresis (SDS-PAGE)

**Figure 3:**
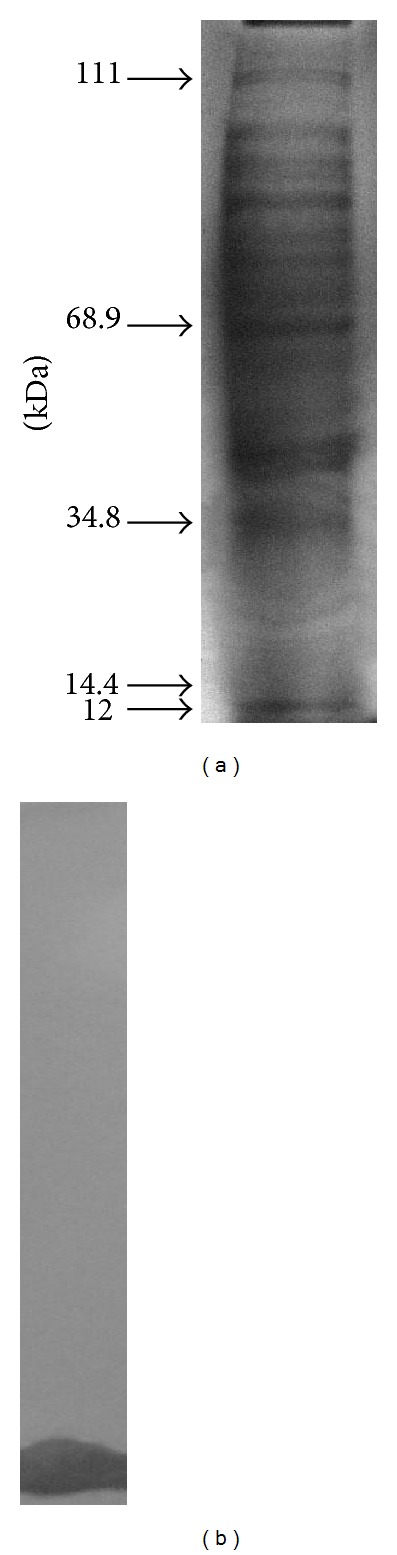
Antimicrobial activity profile presented by strain 235 in SDS-PAGE. The gel strips containing 300-fold concentrated supernatant fluids were stained with Coomassie brilliant blue to reveal the secretory protein profile (a) or overlaid with semisolid LB agar containing the indicator strain (LF-4) to show the inhibition zones (b). For details see text. Numbers on the left indicate relative molecular mass markers (in kDa).

*Streptomyces *sp. 235 was able to secrete polypeptides to the extracellular environment during its growth in a chemically defined medium for 7 days, as demonstrated by SDS-PAGE analysis (Figure 3(a)). The strain released a large amount of extracellular polypeptides with molecular masses varying from 12 to 111 kDa, including one region of the gel (from 12 to 14.4 kDa), which exhibited inhibitory activity when overlaid with the indicator strain *B. pumilus* LF-4 (Figure 3(b)). 

### 3.6. Inhibitory Effect of AMSs against *D. alaskensis*

Experiments concerning the type of activity of AMSs against *D. alaskensis* cells have shown a bacteriostatic effect at a 0.03 g protein/mL (MIC), which corresponded to a 1/4 dilution of the 300-fold concentrated supernatant, whereas a bactericidal effect was observed at a 0.05 g protein/mL (MBC), which corresponded to the 1/2 dilution.

### 3.7. Transmission Electron Microscopy (TEM)

**Figure 4:**
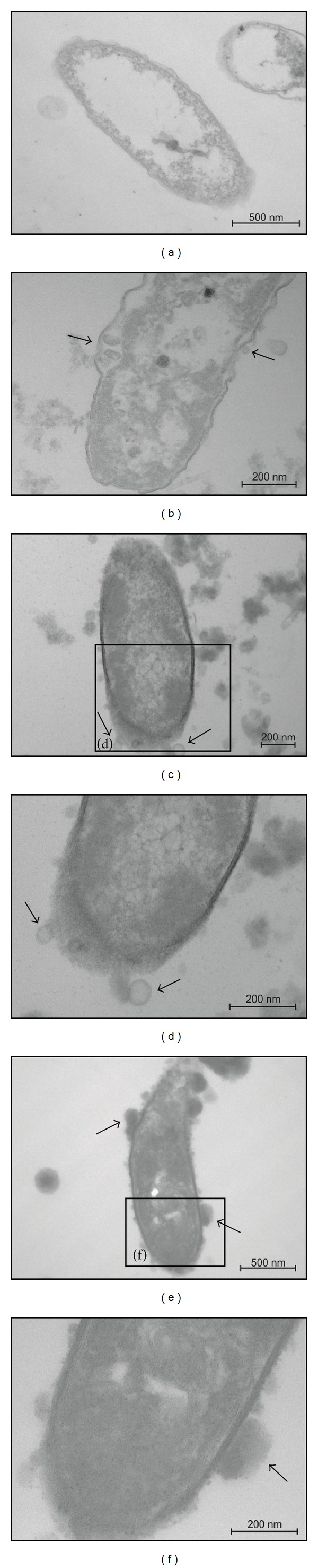
Transmission electron microscopy micrographs of *D. alaskensis* cells. In untreated cells, the inner and outer membranes are visible as continuous and intact structures (a). After treatment with a sub-MIC of AMSs, spherical double-layered structures inside and outside the cell can be observed ((b), black arrow), and the periplasmic space filled with electron-dense material. MIC of AMSs causes the formation of numerous spherical double-layered structures ((c)-(d), black arrows) and the periplasmic space to be filled with electron-dense material. After treatment with a supra-MIC of AMSs, the periplasmic space is still filled with electron-dense material and prominent bubbles are emerging from the cell surface ((e)-(f), black arrows).

Untreated *D. alaskensis* cells in Postgate C medium showed an undamaged structure of the inner membrane and an intact, slightly waved outer membrane, and the periplasmic space was thin with a uniform appearance (Figure 4(a)). *D. alaskensis* cells treated with sub-MIC (0.01 g protein/mL) of the concentrated supernatant of AMSs presented spherical double-layered structures between inner and outer membranes and out of the cell (black arrows in Figure 4(b)). After incubation with a MIC (0.03 g protein/mL) of AMSs, numerous spherical double-layered structures could be observed (black arrow in Figures 4(c)-4(d)). The periplasmic space became completely filled with electron-dense material (Figures 4(c)-4(d)). For a supra-MIC (0.05 g protein/mL) of AMSs, the formation of prominent bubbles emerging from the cell wall surface was observed (black arrows in Figures 4(e)-4(f)), whereas an electron-dense material was still visible in the periplasmic space (Figures 4(e)-4(f)).

## 4. Discussion

Although the four actinomycete strains isolated from the Brazilian soils were able to effectively inhibit the growth of* B. pumilus* LF-4 and *D. alaskensis* NCIMB 13491 *in vitro*, strain 235 was the only one able to inhibit them on solid and in liquid media. This strain was originally isolated from soil of the Atlantic Forest, Vista Chinesa, RJ [23], and characterized as belonging to the *Streptomyces* genus [22]. The molecular characterization by PCR amplification of the *rrs* gene had 99.0% identity with *rrs* gene sequences of *Streptomyces lunalinharesii* RCQ1071. Within the *Streptomyces* genus, the *rrs *gene sequences are highly conserved and sequence identities are high and can be more than 99%. As a result, it becomes very difficult to identify streptomycete species based solely on 16S rDNA sequencing, even when 99.0% similarity to a type strain is observed [45]. Dastager et al. [46] described *Streptomyces deccanensis* as a new *Streptomyces *species even though its *rrs *gene is 99.4% similar to its most closely related type strain. Whole-genome DNA-DNA hybridization remains the “gold standard” to distinguish bacterial species and is necessary to decide whether a strain belongs to a species. When DNA-DNA homology is less than 70%, then the two strains being compared do not belong to the same species. Strain 235 shared around 100% homology with *S. lunalinharesii* RCQ1071, which is far above the 70% threshold recommended for the recognition of separate genomic species [47]. These results have clearly demonstrated that strain 235 indeed belongs to this same species.

*S. lunalinharesii*-type strain RCQ1071^T^ was described recently by our group [44]. It was isolated from another Brazilian soil, in the Central Plateau, under cerrado vegetation [48]. A preliminary study has shown it has the ability to produce antimicrobial substances against human pathogenic microorganisms [22]. Its activity against some phytopathogenic fungi was assessed and the strain was characterized as an excellent chitinase producer [48]. In another study by our group [49], strain 80 from this same species has been shown to be a promising strain for the biological control of *Sclerotinia sclerotiorum*. Here we report on a previously undescribed strain of this species which produces bioactive compounds able to inhibit bacteria involved in the microbial colonization and corrosion of pipe systems in the oil industry.

*S. lunalinharesii* strain 235 was grown in a mineral salt solution containing glucose, pH 7.0, at 28°C for 7 days without shaking. These growth conditions for AMS production are very interesting if we think about future biotechnological application: the use of glucose, a simple, cheap, and easy carbon source along with a mineral salt solution would be very appropriate for an industrial scale production; the use of a neutral pH and fermentation without agitation would be also be very favorable. Most of the methods for antibiotic production described in the literature for *Streptomyces *[50], other bacteria [5], and fungi [51] require similar conditions of carbon source, pH, and temperature. However, they require growth under agitation. Von Der Weid et al. [32] also observed maximum antimicrobial activity when *Paenibacillus peoriae* was cultivated in a chemically defined medium containing glucose at pH 7.0 and incubation at 30°C in stationary conditions.

Peptidic antibiotics, an abundant class of special metabolites, are commonly produced by many microbial species including *Streptomyces*. The preliminary tests for the characterization of AMSs produced by *S. lunalinharesii* 235 have shown that its antimicrobial activity was sensitive to trypsin and proteinase K, indicating its proteic nature. The SDS-PAGE analysis and the presence of an inhibitory zone between 12 and 14.4 kDa have confirmed this finding. This apparent molecular mass range is in accordance with the ultrafiltration membrane approach. However, the AMS was resistant to pronase E suggesting that it is resistant to some types of proteolytic cleavage. The resistance to proteolytic enzymes may indicate the presence of unusual amino acids in the AMS structure. Moreover, the AMS could also present a compact structure, or the lack of cleavage recognition sites, making it resistant to proteolytic enzymes. Cyclic peptides can be resistant to hydrolysis by proteases because their cyclic structure renders them relatively inflexible, which may make cleavage sites inaccessible because of steric hindrance [52]. 

Another interesting characteristic shown by the AMS in this study was its resistance to organic solvents, indicating that its structure should not contain a lipid portion. Also this AMS was able to resist different chemicals and continued to inhibit growth over a range of pH values and high temperatures. Similar characteristics to those of the AMSs produced by *S. lunalinharesii *have been observed for the AMSs produced by other bacterial genera, such as *Bacillus* [5, 53–56], *Paenibacillus* [32, 57], *Lactobacillus* [58], and *Enterococcus *[59]. AMS from *Paenibacillus peoriae*, for instance, presented a high stability after treatments with a broad pH range (3.2–9.6), heat (100°C for 1 h or 121°C for 10 min), proteolytic enzymes and organic solvents, among other tests [32]. *Bacillus subtilis, B. firmus*, and *B. licheniformis*, isolated from an oil reservoir in Brazil, have also produced AMSs that are stable at 100°C for 1 h as well as in presence of other chemicals. In those studies, however, the AMSs were resistant to different proteolytic enzymes and sensitive to several organic solvents, indicating a different chemical nature [5]. *Lactobacillus paracasei *strains have produced an AMS stable at 100°C for only 3 min, and its inhibitory activity was also totally lost after treatment with different proteolytic enzymes [58]. *Enterococcus faecium* was able to produce a bacteriocin which has shown stability at 30°C for 1 h and at 100°C for 30 min in the pH range 2.0–7.0 and 2.0–5.0, respectively, being also sensitive to different proteolytic enzymes [59]. The stability at high temperatures and different pH values, in particular, may be very useful in oil reservoirs, where temperatures above 60°C and pH values ranging between 3.0 and 7.0 are usually found [4].

Cellular alterations caused by AMSs in *D. alaskensis* cells observed by TEM were basically related to the appearance of an electron-dense material in the periplasmic space and to membranes, where spherical double-layered structures appeared and increased from sub-MIC to MIC of AMSs, and prominent bubbles emerge from the cell wall surface at a supra-MIC concentration. Some of these alterations have been observed in other bacterial strains treated with antimicrobial peptides. For instance, Meincken et al. [60] treated *Escherichia coli *cells with the synthetic peptide peptidyl-glycyl-leucine-carboxamide (PGLa) and observed numerous regularly distributed protrusions from cell surface. Latter, Hartmann et al. [61] have treated *E. coli *and* Staphylococcus aureus* cells with gramicidin S, extracted from *Aneurinibacillus migulanus*, or with PGLa, and observed additional intracellular membranous structures and the periplasmic space completely filled with an electron-dense material. They have also observed numerous bubbles which protruded from *E. coli* cell surface.

## 5. Conclusions

Here, for the first time, we report on an antimicrobial substance (AMS) produced by a streptomycete strain, with activity against a sulfate-reducing bacterium *D. alaskensis *NCIMB 13491 and an aerobic bacterium *B. pumilus *LF-4, known to be of importance in corrosion. Strain 235 was identified as belonging to *S. lunalinharesii* species cluster, was originally isolated from a Brazilian soil, and was already known as producer of bioactive compounds against phytopathogenic bacteria and fungi. The AMS, of proteic nature, has shown to be promising for use in oil production plants, given its stability in the presence of several chemicals and solvents, and over a wide range of pH and temperature values. According to TEM, its mechanism of action is probably related to cell membrane alteration and appearance of an electron-dense material in the periplasmic space. The results obtained in this study stimulate further detailed biochemical, molecular, chromatographic, and spectrometric analyses. Having now identified the strain and with the elucidation of the primary structure of the antimicrobial substance, we may have an AMS control agent for sulfate-reducing bacteria commonly found in biofilm and corrosion in the petroleum industry.

## Figures and Tables

**Table 1:** Genus and origin of the actinomycete strains.

Strain	Genus	Origin
221	*Streptomyces* [22]	Soil of the Atlantic Forest (Vista Chinesa—RJ, Brazil) [23]
224	nd^a^	
235	*Streptomyces* [22]	
606	*Streptomyces* [22]	Soil of the Atlantic Forest (Mendanha—RJ, Brazil) [22]

^a^nd: not determined.

**Table 2:** Properties of the antimicrobial substances produced by *Streptomyces  lunalinharesii* 235.

Responses to	Sensitive^b^
Enzymes^a^	
Pronase E	−
Proteinase K	+
Trypsin	+
Solvents (10% and 50%)	
Methanol	−
Ethanol	−
Acetone	−
Chloroform	−
Chemicals	
Urea 6 M	−
NaOH (0.2 M and 1 M)	−
HCl 0.2 M	−
Heat treatment	
40°C for 20, 45 and 60 min	−
60°C for 20, 45 and 60 min	−
80°C for 20, 45 and 60 min	−
100°C for 20, 45 and 60 min	−
Autoclavation (121°C for 20 min)	+
pH^c^	
3.0–6.0	−
7.0–9.0	−

^a^All enzymes were used at final concentration of 1 mg/mL, and tested with 300-fold concentrated supernatant containing the AMS.

^b^(−) inhibition zones similar to those observed in control without treatment, (+) no inhibition zone observed.

^c^pH of supernatants was adjusted from 3.0 to 9.0 varying 1.0 unit before testing their activity.
